# 

**Published:** 1895-12-14

**Authors:** 


					AfcJSUl-U I tLLrY irurcit, tnerexoffP KiiSi,, ueuemhkh iith, isyo.
7 CADBURY S w, CADBURY'S
cocoa m M=s m - COCOA
" The standard of highest purity at present NO ALKALIES USED,
attainable.'?Lanect.
JLf
GLascoiy tVfs t?h/v ~ZZ?*xea. ? >- ^NOAMLHamb NDrwich hosrual \
No. 481. Vol XIX. WITH EXTRA NURSING SUPPLEMENT. pb^^opehcb.
Saturday,
Dec. 14th, 1895.
[Registered at the General Post Office as a Newspaper for Monthly Parts, 6d.; post free, 8d.
transmission in the United Kingdom and Abroad.] Ditto per Annum, 8s.6d., post free.

				

